# Osteopontin and Systemic Lupus Erythematosus Association: A Probable Gene-Gender Interaction

**DOI:** 10.1371/journal.pone.0001757

**Published:** 2008-03-12

**Authors:** Shizhong Han, Joel M. Guthridge, Isaac T. W. Harley, Andrea L. Sestak, Xana Kim-Howard, Kenneth M. Kaufman, Bahram Namjou, Harshal Deshmukh, Gail Bruner, Luis R. Espinoza, Gary S. Gilkeson, John B. Harley, Judith A. James, Swapan K. Nath

**Affiliations:** 1 Oklahoma Medical Research Foundation, Oklahoma City, Oklahoma, United States of America; 2 United States Department of Veterans Affairs Medical Center, Oklahoma City, Oklahoma, United States of America; 3 Department of Medicine, University of Oklahoma Health Sciences Center, Oklahoma City, Oklahoma, United States of America; 4 Louisiana State University Health Science Center, New Orleans, Louisiana, United States of America; 5 Medical University of South Carolina, Charleston, South Carolina, United States of America; Leiden University Medical Center, Netherlands

## Abstract

Osteopontin (*SPP1*) is an important bone matrix mediator found to have key roles in inflammation and immunity. *SPP1* genetic polymorphisms and increased osteopontin protein levels have been reported to be associated with SLE in small patient collections. The present study evaluates association between SPP1 polymorphisms and SLE in a large cohort of 1141 unrelated SLE patients [707 European-American (EA) and 434 African-American (AA)], and 2009 unrelated controls (1309 EA and 700 AA). Population-based case-control association analyses were performed. To control for potential population stratification, admixture adjusted logistic regression, genomic control (GC), structured association (STRAT), and principal components analysis (PCA) were applied. Combined analysis of 2 ethnic groups, showed the minor allele of 2 SNPs (rs1126616T and rs9138C) significantly associated with higher risk of SLE in males (P = 0.0005, OR = 1.73, 95% CI = 1.28–2.33), but not in females. Indeed, significant gene-gender interactions in the 2 SNPs, rs1126772 and rs9138, were detected (P = 0.001 and P = 0.0006, respectively). Further, haplotype analysis identified rs1126616T-rs1126772A-rs9138C which demonstrated significant association with SLE in general (P = 0.02, OR = 1.30, 95%CI 1.08–1.57), especially in males (P = 0.0003, OR = 2.42, 95%CI 1.51–3.89). Subgroup analysis with single SNPs and haplotypes also identified a similar pattern of gender-specific association in AA and EA. GC, STRAT, and PCA results within each group showed consistent associations. Our data suggest *SPP1* is associated with SLE, and this association is especially stronger in males. To our knowledge, this report serves as the first association of a specific autosomal gene with human male lupus.

## Introduction

Systemic Lupus Erythematosus (SLE) is a prototypic human autoimmune disease characterized by impaired T cell responses, dysregulated B cell activation, hyperactive B cells and autoantibody production leading to inflammation and potential end-organ damage. While the etiology of SLE remains complex, genetic factors are known to be important in the pathogenesis of SLE [1], [2]. The current collection of genetic information suggests that SLE susceptibility arises from specific combinations of multiple gene-gene and gene-environment interactions. Among the genetic factors believed to influence SLE susceptibility, the major histocompatibility complex (MHC) alleles show the most significant association, but these do not explain the total genetic background of the disease. Importantly, several recent studies show that non-HLA genes play a role in SLE development [3]–[7]. Recently, several lines of evidence suggest that secreted phosphoprotein 1 (*SPP1*) located at 4q22, also called osteopontin and early T-lymphocyte activation 1, may have a role in the pathogenesis of SLE as well as other autoimmune disorders.

*SPP1* plays a key role in bone biology and has recently found to also be important in regulating inflammation and immunity. The immunologic functions of *SPP1* include enhancing the proinflammatory Th1 cell response and inhibiting the Th2 responses [8]–[9]. In addition, some studies have suggested that *SPP1* plays a role in the survival of activated T cells by inducing apoptosis, while others have demonstrated the essential role of an intracellular form of *SPP1* in the production of interferon-alpha by plasmacytoid dendritic cells [10], [11]. Humans with SLE and autoimmune prone mice over express osteopontin suggesting that abnormal expression of this protein may participate in SLE disease pathogenesis [12], [13]. Further, polymorphic osteopontin alleles have been implicated in the development of a mouse model of lupus [14].

SNPs in the *SPP1* gene have also been reported to be associated with human SLE, adding further support to the role of this gene in SLE pathogenesis [15]. A significant association between rs11226616 and SLE was first demonstrated in a small North American Caucasian cohort study [15]. Two SNPs (rs1126772 and rs9138) in the 3′ UTR in the *SPP1* gene were associated with high levels of *SPP1* and elevated risk of developing autoimmune/lymphoproliferative syndrome (ALPS), a disorder which leads to an autoimmune pattern similar to lupus prone strains of mice [16]. The same group later showed significant associations between SLE and 2 *SPP1* SNPs (rs7687316 and rs9138) in an Italian population [17]. This information prompted us to test association between *SPP1* polymorphisms and SLE in a large, multi-ethnic collection.

## Results

Marker information, minor allele frequency and the statistical significance for allele distributions between cases and controls are presented in Table 1. The only significant difference in allele distribution was observed in the combined male-female group for rs6840362, which showed a significant difference allele distribution in EA (P = 0.015). However, significant differences were evident in the male subgroup. This finding was especially strong in EA males, where 3 SNPs (rs1126616, rs1126772 and rs9138) showed significant differences in allele distributions. Similarly, in AA males, 2 SNPs (rs1126616 and rs9138) demonstrated significant differences in allele distribution.

**Table 1:** Marker information and minor allele frequency in African-American and European-American sample.

					African-American sample				European-American sample			
					434/700†				707/1309†			
					F: 403/475; M: 31/225†				F: 617/936; M: 90/373†			
SNP	Position	Type	Alleles	STRATA		Case	Control			Case	Control	
					MA	MAF	MAF	P*	MA	MAF	MAF	P*
rs2728127	89252294	5′ near gene	G/A	All	A	0.476	0.475	0.97	G	0.296	0.288	0.59
				Female	A	0.471	0.496	0.31	G	0.301	0.284	0.31
				Male	A	0.533	0.431	0.13	G	0.261	0.298	0.33
rs2853744	89253427	5′ near gene	G/T	All	T	0.252	0.240	0.52	T	0.060	0.058	0.79
				Female	T	0.252	0.240	0.57	T	0.061	0.060	0.88
				Male	T	0.258	0.241	0.77	T	0.050	0.052	0.90
rs11730582	89253600	5′ near gene	T/C	All	C	0.141	0.131	0.49	C	0.477	0.502	0.12
				Female	C	0.147	0.138	0.59	C	0.472	0.500	0.12
				Male	C	0.065	0.116	0.22	T	0.489	0.493	0.92
rs2853749	89254993	intron_0	C/T	All	T	0.393	0.393	0.98	T	0.294	0.289	0.71
				Female	T	0.392	0.387	0.84	T	0.299	0.285	0.38
				Male	T	0.403	0.406	0.96	T	0.261	0.299	0.32
rs11728697	89256120	intron_3	C/T	All	T	0.244	0.230	0.44	C	0.423	0.412	0.50
				Female	T	0.238	0.242	0.85	C	0.429	0.412	0.34
				Male	T	0.323	0.204	**0.03**	C	0.382	0.413	0.45
rs6840362	89257099	intron_3	C/T	All	T	0.354	0.337	0.42	T	0.258	0.295	**0.01**
				Female	T	0.354	0.330	0.29	T	0.259	0.285	0.11
				Male	T	0.355	0.353	0.98	T	0.256	0.322	0.08
rs6811536	89259584	intron_4	C/T	All	T	0.418	0.405	0.53	T	0.280	0.307	0.07
				Female	T	0.413	0.399	0.56	T	0.281	0.297	0.32
				Male	T	0.500	0.418	0.23	T	0.272	0.332	0.12
rs10516799	89260372	intron_5	G/C	All	C	0.344	0.328	0.44	C	0.279	0.308	0.05
				Female	C	0.346	0.324	0.32	C	0.279	0.298	0.26
				Male	C	0.317	0.338	0.74	C	0.272	0.332	0.12
rs1126616	89261032	exon_6	C/T	All	T	0.209	0.185	0.17	T	0.288	0.270	0.22
				Female	T	0.202	0.190	0.50	T	0.280	0.285	0.79
				Male	T	0.290	0.176	**0.03**	T	0.344	0.235	**0.002**
rs1126772	89261365	3′ UTR	A/G	All	G	0.053	0.061	0.45	G	0.220	0.218	0.86
				Female	G	0.056	0.061	0.65	G	0.214	0.229	0.31
				Male	G	0.016	0.060	0.15	G	0.267	0.190	**0.02**
rs9138	89261521	3′ UTR	A/C	All	C	0.205	0.184	0.23	C	0.286	0.271	0.31
				Female	C	0.198	0.188	0.60	C	0.277	0.285	0.64
				Male	C	0.290	0.176	**0.03**	C	0.344	0.235	**0.002**

MAF = minor allele frequency;

MA = minor allele;

* = chi-square test or Fisher exact test where appropriate, significant associations at the 0.05 significance level are bold.

† case/control number in all, male and female subgroup respectively.

We evaluated the association of each polymorphism with SLE, adjusting for the admixture proportion utilizing logistic regression under the multiplicative genetic model for minor alleles by combined analysis. Considering the gender effect in allele distributions and possible race specific effect in disease susceptibility, subgroup analysis stratified by gender and race were also performed. Table 2 shows the association results in detail. Briefly, 2 SNPs' minor alleles (rs1126616T and rs9138C) showed significant associations with SLE in AA and EA combined males, but not in females, both of which conferred a high risk of SLE (P = 0.0005, OR = 1.73, 95%CI 1.28–2.33). Indeed, significant gene-gender interactions in the 2 SNPs, rs1126772 and rs9138, were detected (P = 0.001, P = 0.0006, respectively). Subgroup analysis by race revealed the same trend in AA and EA. For AA, 3 SNPs (rs11728697, rs1126616, and rs9138) showed significant association in males only (P = 0.02, P = 0.027, P = 0.027, respectively). In EA males, 3 SNPs (rs1126616, rs1126772, and rs9138) also showed significant association (P = 0.003, P = 0.028, P = 0.003). Furthermore, to exclude the false positive association which can arise from hidden population substructure, we utilized GC, STRAT, and PCA to verify the association result in each population. All associations remained consistent with the admixture adjusted logistic regression analysis (Table 3).

**Table 2:** Associations of SNPs with SLE by logistic regression under multiplicative genetic model

SNP	Strata	AA samples		EA samples		Combined sample	
		P	OR(95% CI)	P	OR(95% CI)	P	OR(95% CI)
rs2728127	All	0.72	0.97 (0.81–1.16)	0.59	1.04 (0.90–1.20)	0.56	1.03 (0.92–1.16)
	Female	0.34	0.91 (0.75–1.10)	0.29	1.09 (0.93–1.28)	0.16	1.09 (0.97–1.23)
	Male	0.10	1.56 (0.91–2.68)	0.35	0.84 (0.59–1.21)	0.10	0.78 (0.57–1.05)
rs2853744	All	0.61	1.05 (0.86–1.29)	0.9	1.02 (0.77–1.34)	0.65	1.04 (0.88–1.22)
	Female	0.62	1.06 (0.85–1.32)	0.85	1.03 (0.77–1.38)	0.61	1.05 (0.88–1.25)
	Male	0.98	1.01 (0.56–1.82)	0.92	0.96 (0.46–2.01)	0.90	1.03 (0.65–1.63)
rs11730582	All	0.63	1.07 (0.82–1.40)	0.17	0.91 (0.80–1.04)	0.35	0.95 (0.84–1.06)
	Female	0.44	1.12 (0.84–1.48)	0.12	0.89 (0.77–1.03)	0.29	0.93 (0.82–1.06)
	Male	0.35	0.62 (0.21–1.80)	0.99	1.00 (0.73–1.37)	0.78	0.96 (0.71–1.29)
rs2853749	All	0.92	1.01 (0.84–1.21)	0.69	1.03 (0.89–1.19)	0.72	1.02 (0.91–1.14)
	Female	0.87	1.02 (0.84–1.23)	0.36	1.08 (0.92–1.26)	0.42	1.05 (0.93–1.19)
	Male	0.87	0.96 (0.56–1.64)	0.33	0.84 (0.58–1.20)	0.39	0.88 (0.65–1.18)
rs11728697	All	0.44	1.09 (0.88–1.35)	0.57	1.04 (0.91–1.19)	0.97	1.00 (0.89–1.12)
	Female	0.95	0.99 (0.79–1.25)	0.33	1.07 (0.93–1.24)	0.40	0.95 (0.84–1.07)
	Male	**0.02**	**2.03 (1.13–3.63)**	0.53	0.90 (0.65–1.25)	0.10	1.28 (0.95–1.71)
rs6840362	All	0.33	1.10 (0.91–1.32)	**0.03**	**0.85 (0.73–0.99)**	0.30	0.94 (0.84–1.06)
	Female	0.29	1.11 (0.91–1.36)	0.10	0.87 (0.74–1.03)	0.56	0.96 (0.85–1.09)
	Male	0.86	0.95 (0.55–1.63)	0.06	0.70 (0.48–1.02)	0.15	0.80 (0.59–1.09)
rs6811536	All	0.37	1.09 (0.91–1.31)	0.12	0.89 (0.77–1.03)	0.52	0.96 (0.86–1.08)
	Female	0.57	1.06 (0.87–1.28)	0.31	0.92 (0.78–1.08)	0.67	0.97 (0.86–1.10)
	Male	0.31	1.33 (0.77–2.30)	0.09	0.73 (0.51–1.06)	0.49	0.90 (0.67–1.21)
rs10516799	All	0.41	1.08 (0.90–1.31)	0.09	0.88 (0.76–1.02)	0.43	0.96 (0.85–1.07)
	Female	0.32	1.11 (0.91–1.35)	0.25	0.91 (0.78–1.07)	0.78	0.98 (0.87–1.11)
	Male	0.68	0.89 (0.5–1.56)	0.09	0.73 (0.51–1.06)	0.14	0.80 (0.59–1.08)
rs1126616	All	0.16	1.18 (0.94–1.47)	0.34	1.07 (0.93–1.25)	0.13	1.10 (0.97–1.25)
	Female	0.48	1.09 (0.86–1.39)	0.81	0.98 (0.83–1.15)	0.84	1.01 (0.89–1.16)
	Male	**0.027**	**2.01 (1.10–3.69)**	**0.003**	**1.71 (1.2–2.44)**	**0.0005**	**1.73 (1.28–2.33)**
rs1126772	All	0.45	0.86 (0.59–1.27)	0.98	1.00 (0.85–1.17)	0.73	0.97 (0.84–1.13)
	Female	0.70	0.93 (0.62–1.38)	0.30	0.91 (0.76–1.09)	0.27	0.91 (0.78–1.07)
	Male	0.19	0.32 (0.04–2.44)	**0.028**	**1.54 (1.05–2.26)**	0.10	1.36 (0.95–1.95)
rs9138	All	0.20	1.16 (0.93–1.45)	0.44	1.06 (0.91–1.23)	0.19	1.09 (0.96–1.23)
	Female	0.58	1.07 (0.84–1.36)	0.65	0.96 (0.82–1.13)	0.96	1.00 (0.87–1.14)
	Male	**0.027**	**2.01 (1.10–3.69)**	**0.003**	**1.71 (1.2–2.44)**	**0.0005**	**1.73 (1.28–2.33)**

Significant associations at the 0.05 significance level are bold. For Combined sample, P value and OR were adjusted for admixture proportion and gender; for each gender-specific sample, only admixture proportion was used as a covariate

**Table 3:** Associations of SNPs with SLE by genomic control (GC), structured association test (STRAT), and principle components analysis (PCA).

SNP	Strata	African-American sample			European-American sample		
		GC	STRAT	PCA	GC	STRAT	PCA
rs2728127	ALL	0.96	0.48	0.83	0.57	0.78	0.63
	Female	0.31	0.67	0.31	0.29	0.18	0.38
	Male	0.13	0.20	**0.03**	0.38	0.25	0.23
rs2853744	ALL	0.55	0.72	0.67	0.77	0.89	0.80
	Female	0.55	0.65	0.25	0.85	0.93	0.91
	Male	0.91	0.88	0.85	0.91	0.92	0.71
rs11730582	ALL	0.49	0.78	0.29	0.12	0.15	0.13
	Female	0.60	0.59	0.59	0.11	0.06	0.20
	Male	0.32	0.32	0.48	0.92	0.99	0.88
rs2853749	ALL	0.93	0.50	0.83	0.69	0.88	0.75
	Female	0.83	0.66	0.73	0.36	0.22	0.43
	Male	0.75	0.96	0.63	0.37	0.24	0.22
rs11728697	ALL	0.43	0.70	0.24	0.48	0.67	0.56
	Female	0.83	0.79	0.79	0.33	0.44	0.49
	Male	**0.033**	0.095	**0.005**	0.46	0.61	0.32
rs6840362	ALL	0.45	0.73	0.53	**0.012**	**0.011**	**0.013**
	Female	0.30	0.57	0.65	0.11	0.07	0.12
	Male	0.96	0.26	0.75	0.11	0.07	0.14
rs6811536	ALL	0.55	0.81	0.62	0.06	0.06	0.07
	Female	0.58	0.77	0.29	0.31	0.41	0.36
	Male	0.29	0.25	0.37	0.15	0.099	0.18
rs10516799	ALL	0.47	0.75	0.51	**0.049**	**0.047**	0.054
	Female	0.34	0.66	0.56	0.25	0.34	0.30
	Male	0.70	0.19	0.61	0.15	0.10	0.18
rs1126616	ALL	0.16	0.19	0.11	0.21	0.34	0.18
	Female	0.51	0.82	0.31	0.81	0.87	1.00
	Male	**0.046**	0.084	**0.016**	**0.006**	**0.002**	**0.004**
rs1126772	ALL	0.47	0.72	0.55	0.88	0.98	0.83
	Female	0.65	0.89	0.62	0.29	0.30	0.48
	Male	0.26	0.19	0.30	**0.039**	**0.025**	**0.032**
rs9138	ALL	0.22	0.25	0.15	0.29	0.47	0.26
	Female	0.61	0.89	0.54	0.65	0.79	0.83
	Male	**0.046**	0.079	**0.016**	**0.006**	**0.002**	**0.004**

Significant associations at the 0.05 significance level are bold.

To assess the effect of any particular clinical feature on the genetic association, we have performed a subgroup analysis classified by eight clinical characteristics (Cutaneous manifestations, arthritis, serositis, renal involvement, neuro-psychiatric manifestations, hematological features, anti-dsDNA, antinuclear antibody) available for males (91 male SLE) and females (754 female SLE) separately. Although we did not find any significant association in females, we did observed evidence of association in males for some of the clinical features; however, these associations are not stronger than the overall male-specific analysis. Therefore, there seems to be no evidence that the overall effect is dominated by a particular subset.

**Figure 1:**
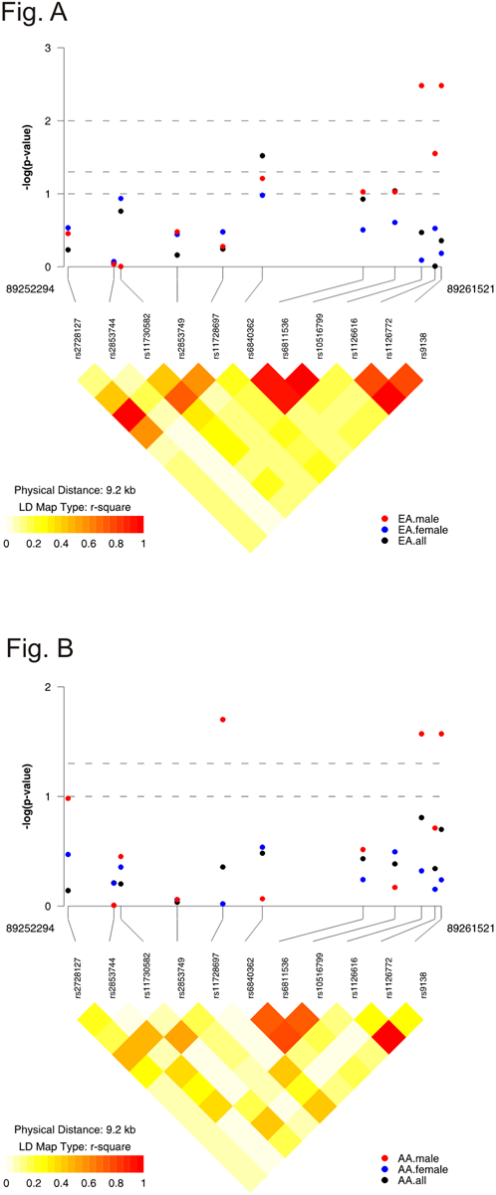
Linkage disequilibrium (LD) structure and association P value in AA. LD is calculated from the genotype data in AA healthy control population. Fig. 1B: LD structure and association P value in EA. LD is calculated from the genotype data in EA healthy control population.

Haplotype analysis was performed to further evaluate the role of *SPP1* in SLE susceptibility. We conducted haplotype reconstruction and linkage disequilibrium analysis incorporating all 11 SNPs in the *SPP1* gene. The LD map and each SNP associated P value are depicted in Fig. 1. Notably, rs1126616 and rs9138 are almost in complete LD in both AA (Fig. 1A) and EA (Fig. 1B), which explained their similar behavior in the association with SLE. Initially, we included all selected 11 SNPs for haplotype analysis in combined data and each race specific population adjusted by admixture proportion. In accordance with the results of single SNP analysis, no significant global association was detected either in the combined analysis or in each specific population. The same trend remained in the female subgroup. However, significant haplotype association was observed in the male subgroup for the combined, AA and EA (P = 0.001, P = 0.003, P = 0.028, respectively).

To further explore the haplotype effect in male subgroup, we performed conditional analysis to detect if there was a specific SNP or a subset of SNPs which can explain the global haplotype association. Interestingly, the last 3 SNPs (rs1126616, rs1126772, and rs9138) explained the whole association for each significant group. When we performed haplotype association analysis conditional on these 3 SNPs, all significant associations disappear in the combined AA and EA subgroup. We next focused on these 3 SNPs for haplotype analysis. Detailed results are presented in Table 4. Although single SNP analysis revealed only marginal significant association, it is notable that in AA males the haplotype analysis demonstrated a much stronger association (P = 0.002). Specifically, the haplotype TAC confers a high risk of SLE in AA males (P = 0.001, OR = 3.37, 95%CI 1.65–6.92). In EA males, the TAC haplotype is not significant, but there is a trend in the same direction towards association with SLE (P = 0.079, OR = 1.84, 95%CI 0.96–3.53). Furthermore, in the combined male subgroup, the haplotype TAC significantly increases the risk of SLE (P = 0.0004, OR = 2.42, 95%CI 1.51–3.89). All the association P-values were also verified by 10,000 permutations. Accordingly, interaction analysis between haplotype and gender also showed significant interactions in AA, EA, and the combined analysis (P = 0.018, P = 0.02, P = 0.0018, respectively).

**Table 4:** Haplotypic analysis of SPP1 polymorphisms (rs1126616, rs1126772, and rs9138) and SLE risk.

Sample	Strata (Case/Control)	Haplotype	Case	Control	P	OR (95%CI)	Global P	Permutation*	Interaction
AA	All (434/700)	TAC	0.155	0.124	**0.03**	1.33 (1.03–1.73)	0.079	0.077	
	Female (403/475)	TAC	0.145	0.128	0.29	1.16 (0.88–1.52)	0.53	0.53	**0.018**
	Male (31/225)	TAC	0.274	0.116	**0.001**	3.37 (1.65–6.92)	**0.002**	**0.002**	
EA	All (707/1309)	TAC	0.068	0.052	0.062	1.30 (0.99–1.70)	0.17	0.18	
	Female (617/936)	TAC	0.067	0.055	0.21	1.21 (0.90–1.63)	0.31	0.3	**0.02**
	Male (90/373)	TAC	0.078	0.044	0.079	1.84 (0.96–3.53)	**0.01**	**0.01**	
Combined	All (1141/2009)	TAC	0.101	0.077	**0.006**	1.30 (1.08–1.57)	**0.022**	**0.02**	
	Female (1020/1411)	TAC	0.098	0.080	0.11	1.18 (0.97–1.44)	0.18	0.18	**0.0018**
	Male (121/598)	TAC	0.128	0.071	**0.0004**	2.42 (1.51–3.89)	**0.0003**	**0.0007**	

Significant associations at the 0.05 significance level are bold.

* Significance was assessed by 10,000 permutations

For Combined sample, P value and OR were adjusted for admixture proportion and gender; for each gender-specific sample, only admixture proportion was used as a covariate

## Discussion

Our study confirms the previously reported genetic association with SLE and presents additional support in a large multiethnic cohort. Previous studies have suggested that increased *SPP1* plasma concentration, as a result of increased gene/protein expression and local production, was associated with SLE [13]. Therefore, *SPP1* is a reasonable candidate gene for SLE susceptibility. In the first report, Forton et al found that a silent polymorphism (rs1126616) in exon 7 was significantly associated with SLE [15]. Subsequently, 2 SNPs in the 5′ (rs7687316) and 3′ (rs9138) ends of the *SPP1* gene were reported to contribute to SLE susceptibility [16]. Although our combined male-female results were marginally significant, associations with SLE were found for haplotype analysis of 3 SNPs (rs1126616, rs1126772, and rs9138). Since significant interactions were detected between gender and the *SPP1* SNPs, haplotypes were analyzed separately for males and females. Our unique findings focus on stronger associations found in male SLE patients from the combined analysis of samples from 2 different ethnic populations. Haplotype analyses revealed that the last 3 SNPs in 3′UTR explain the global association in males and supports the hypothesis that the causal variant of *SPP1* might be near 3′UTR, which could affect the expression level of *SPP1*.

SLE is at least nine times more prevalent in female compared to male subjects; however, the underlying cause of the gender effect has not been clearly established. There is evidence that gender-specific genetic effects exist, both from the many differences in animal models of lupus and from other previous work in humans [18]–[21]. In a recent study by our group [22], a SLE linkage at 13q32 was identified and replicated by restricting the samples to a relatively homogenous population of African-American families containing at least one male affected. Therefore, taken all the evidences together, the evolving data suggest unique clinical courses and genetic predispositions in male lupus. Further study is warranted to see if differences in osteopontin regulation are involved in these differences.

Our haplotype analyses findings could also be significant due to possible functionality of the three 3′UTR SNPs. Recent years have seen an increased appreciation for the importance of post-transcriptional regulation in eukaryotic organisms [23]. The untranslated region at the 3′ end of a gene (3′UTR) is involved in regulating gene expression at both the pre-mRNA level and the mature mRNA level. In the former, the 3′UTR plays a central role in mRNA 3′ processing and polyadenylation, whereas in the latter cis-elements in the 3′UTR are bound by trans-acting factors which modulate mRNA stability, nuclear export, subcellular localization, and translation efficiency [24]–[27]. Micro-RNA target sites in 3′UTRs have been shown to be highly conserved [28]. Polymorphisms in microRNA target sites within the 3′UTR may influence gene expression in complex phenotypes, such as lupus [29]. 3′UTRs are also preferred sites of cis-encoded natural antisense transcripts [30]. A search of the PolyA_DB2 database shows that there is supporting cDNA/EST evidence for 2 transcripts with different polyadenylation sites in the SPP1 gene [31]. In addition, the PolymiRTS (polymorphic microRNA Target Site) database revealed evidence that suggests that SNP rs1126772, which shows association with lupus in this report, is predicted to disrupt a non-conserved microRNA target site [32]. Two 3′UTR SNPs, rs1126893, and rs2853754, which are not tested in the present association study, are predicted to disrupt two evolutionarily conserved microRNA target sites. Interestingly, the rs1126893 is in complete LD and within the same haplotype block with our associated SNPs (rs1126772 and rs9138 at the 3′UTR) based on Hapmap data of CEPH families. In fact, the distance between rs1126893 and rs2853754 is only 30 bp, the alternative allele for SNP rs2853754 is predicted to create microRNA target sites for 2 microRNAs. A more extensive analysis of these potential mechanisms for the 3′UTR control of *SPP1* gene expression will be required to determine if these predicted sites may be responsible for this genetic association.

However, we acknowledge that the current study suffers from some shortcomings. First, the samples size for the male SLE patients is small (121 males); the association results in male subgroup analysis might arise by chance. However, the possibility of false positives arising from the multiple testing problems should be small since the reported P value is significant enough to survive even the most conservative Bonferroni correction. Second, none of the previously published functional studies were performed to evaluate the role of *SPP1* in males and females separately. While this experiment is beyond the scope of the present study, we hope to assess *SPP1* gene and protein expression in male and female lupus patients in future. It is worth noting that sex-specific differences in *SPP1* gene expression have been observed in rats [33], [34]. Third, an independent replication study should be performed to further verify the male association. Like many other association studies with complex phenotype, no matter how large, validation in a second cohort is needed.

In summary, our data suggest the SPP1 gene might be associated with the development of SLE in general and especially in males. To our knowledge, this report serves as the first description of a gender-specific human lupus genetic association. Further in-depth molecular, genetic, and functional studies should improve our understanding of the disease, and hopefully will provide a more accurate diagnostic algorithm and improved genetic counseling and management strategies.

## Materials and Methods

### Patients and controls

Genomic DNA samples from SLE patients and control subjects were collected after obtaining written, informed consent from 1141 unrelated SLE patients [707 European-American (EA), 434 African-American (AA)], including 121 males (31 AA male patients and 90 EA male patients), and 2009 unrelated controls (1309 EA, 700 AA), including 598 males (225 AA male controls and 373 EA male controls). Coded DNA samples were obtained from the Lupus Family Registry and Repository (LFRR: http://lupus.omrf.org/). The study was approved by the Institutional Review Boards (IRB) at the Oklahoma Medical Research Foundation and University of Oklahoma Health Sciences Center. All patients utilized in the study met SLE classification based upon the revised criteria of the American College of Rheumatology (ACR) [35].

### Genotyping

Initially, we evaluated 32 SNPs in the *SPP1* gene on the Illumina SNP platform (San Diego, CA) at the University of Texas Southwestern Microarray Core Facility (Dallas, TX) using standard methods described in more detail (www.illumina.com). A summary of the genotyping information for all the markers is shown in supplementary Table S1. We included 11 SNPs in the final analysis in which the controls in Hardy-Weinberg equilibrium, and a minor allele frequency of >1% in both the populations. Marker information, genotype, and allele distributions of the 11 SNPs in cases and controls are summarized in supplementary Table S2. Of the SNPs that failed quality control, 13 SNPs were monomorphic in 2 studied populations, 6 SNPs had minor allele frequencies which were below 1% at least in one population, 2 SNPs failed genotyping, and 1 SNP deviated significantly from HWE in the black control group.

To know how much of the genetic variation of the SPP1 gene that is captured by the 11 successfully genotyped SNPs, we downloaded the tagged SNPs of SPP1 from the CEU population from Hapmap database (http://www.hapmap.org) using Tagger Pairwise method, in which MAF and LD (r-squared) cutoffs were set to 0.05 and 0.8, respectively. We found 4 tagged SNPs, rs6840362, rs9138, rs2853749, and rs11728697, which can capture all other 10 genotyped SNPs by Hapmap project across the gene. Actually, all these 4 tag SNPs were included in our analyzed marker list. Therefore, all the known genetic variation of the SPP1 gene should be captured by the 11 SNPs we analyzed, especially for the European population.

### Statistical analysis

Departures from Hardy–Weinberg equilibrium in cases and controls were tested for SNPs with a Pearson Chi-square test. Allele frequencies in SLE cases were compared to those in control subjects using the Chi-square test or Fisher's exact probability test, where appropriate. Statistical evaluations for testing genetic effects were performed using multivariate logistic regression analysis with adjustments for gender and admixture proportions under multiplicative genetic model. Interaction analysis was performed by introducing the interaction term into the logistic regression model including admixture proportion as a covariate. Statistical significance was obtained by the likelihood ratio test comparing the models with and without the interaction term. Haplotype-based association analysis and conditional analysis were performed by the WHAP program [36].

To control for possible confounding due to population stratification, a panel of 221 ancestry informative markers (AIMs) was genotyped using the same Illumina SNP platform for an ongoing association study project in these LFRR samples. Frequencies of the AIMS in the ancestry population European and African are shown in supplementary Table S3. These AIMs were selected based on the criteria of large allele frequency differences (20% or greater allele frequency difference) between 2 ancestral populations, HWE in ancestral populations and all 221 AIMs were separated by al least 1 cM to minimize the possibility of strong LD between AIMs. We used the STRUCTURE program to estimate the admixture proportion for each individual in AA and EA separately [37]. The log likelihood of each analysis at varying number of population groups (*k*) was estimated from the average of 3 independent runs (20,000 burn in and 30,000 iterations). As expected, the results favored a two-ancestry population model in both AA and EA. The average proportion of European ancestry was 0.17 in AA samples and 0.99 in EA samples. We included the European ancestry proportion in each individual as a covariate in the logistic regression model to control the population stratification in subgroup and combined analysis. Additionally, genomic control (GC) [38], structured association (STRAT) [39], and principal components analysis (PCA) [40] were applied to control for population stratification in race specific analysis.

## Supporting Information

Table S1 (0.02 MB XLS)

Table S2 (0.02 MB XLS)

Table S3 (0.05 MB XLS)
